# BRCA2 mutation in advanced lung squamous cell carcinoma treated with Olaparib and a PD-1 inhibitor: a case report

**DOI:** 10.3389/fonc.2023.1190100

**Published:** 2023-05-16

**Authors:** Zhujun Chen, Kang Wang, Lintao Zhao, Liang Gong

**Affiliations:** Department of Respiratory and Critical Care Medicine, The First Affiliated Hospital of Army Medical University, Chongqing, China

**Keywords:** Olaparib, lung squamous cell carcinoma, PD-1 inhibitor, BRCA2 mutation, case report

## Abstract

**Background:**

Mutations in the human breast cancer susceptibility gene 2 (breast cancer 2, BRCA2) increase the risk of breast, ovarian and other cancers. Olaparib, an oral poly[adenosine diphosphate (ADP)–ribose] polymerase (PARP) inhibitor, is usually prescribed to treat BRCA mutated tumors, especially breast and ovarian cancers. Programmed cell death-1 (PD-1) inhibitors have revolutionized the treatment of lung cancer and many other cancers by destroying the interaction between receptors with ligands in the tumor-immune microenvironment and enabling T cells to recognize and attack cancer cells.

**Case description:**

In our study, we report a patient with advanced BRCA2 lung squamous cell carcinoma who received platinum-based chemotherapy combined with paclitaxel. Seven months later, the disease progressed. BRCA2 mutations were detected in peripheral blood by next-generation sequencing. After 2 months of treatment with Olaparib combined with Cindilimab, the patient was in partial remission and the progression-free survival (PFS) lasted for 6 months, but the patient developed immune renal damage.

**Conclusions:**

This study adds to the clinical data for the treatment of BRCA2 mutant non-small cell lung cancer by demonstrating that lung squamous cell carcinoma has a good response to PARP inhibitors. It also serves as a reminder that there may still be some negative effects from targeted superimposed immunotherapy.

## Introduction

The BRCA tumor suppressor gene family, which consists of BRCA1 and BRCA2, preserves genomic stability by encoding a protein involved in the deoxyribonucleic acid (DNA) homologous recombination and reparation mechanism (1). BRCA2 mutations raise the risk of several cancers, including breast and ovarian cancers in women and prostate cancer in men (2, 3). In addition, it was found that homologous recombination (HR) repair genes, including BRCA2, may be related with lung carcinogenesis (4). DNA double-strand damage repair depends on HR and non-homologous end joining (NHEJ) repair recombination pathways, BRCA2 plays an important role in the HR pathway. PARP inhibitors can block the repair of single-stranded DNA by PARP and cause double-stranded NDA breaks during replication. Cancer cells with BRCA2 mutations are unable to repair double-stranded DNA through the HR pathway and eventually die (5). Clinical trials (6–9) have revealed that the PARP inhibitor Olaparib has considerable effects on the breast, ovarian, prostate, and pancreatic cancer species, but there is still insufficient proof to support its use in the treatment of lung squamous cell carcinoma. Here, we provide a case of a patient with lung squamous cell carcinoma who had make progressive disease after prior two-drug chemotherapy. Next, the BRCA2 mutation was detected by blood next-generation sequencing, and the PFS lasted for 6 months after the administration of PARP inhibitor (Orapalil) and a PD-1 inhibitor. This may suggest that we can choose Olaparib-targeted therapy for patients with BRCA2 mutated non-small cell lung cancer, especially for patients with lung squamous cell carcinoma.

## Case presentation

**Figure 1:**
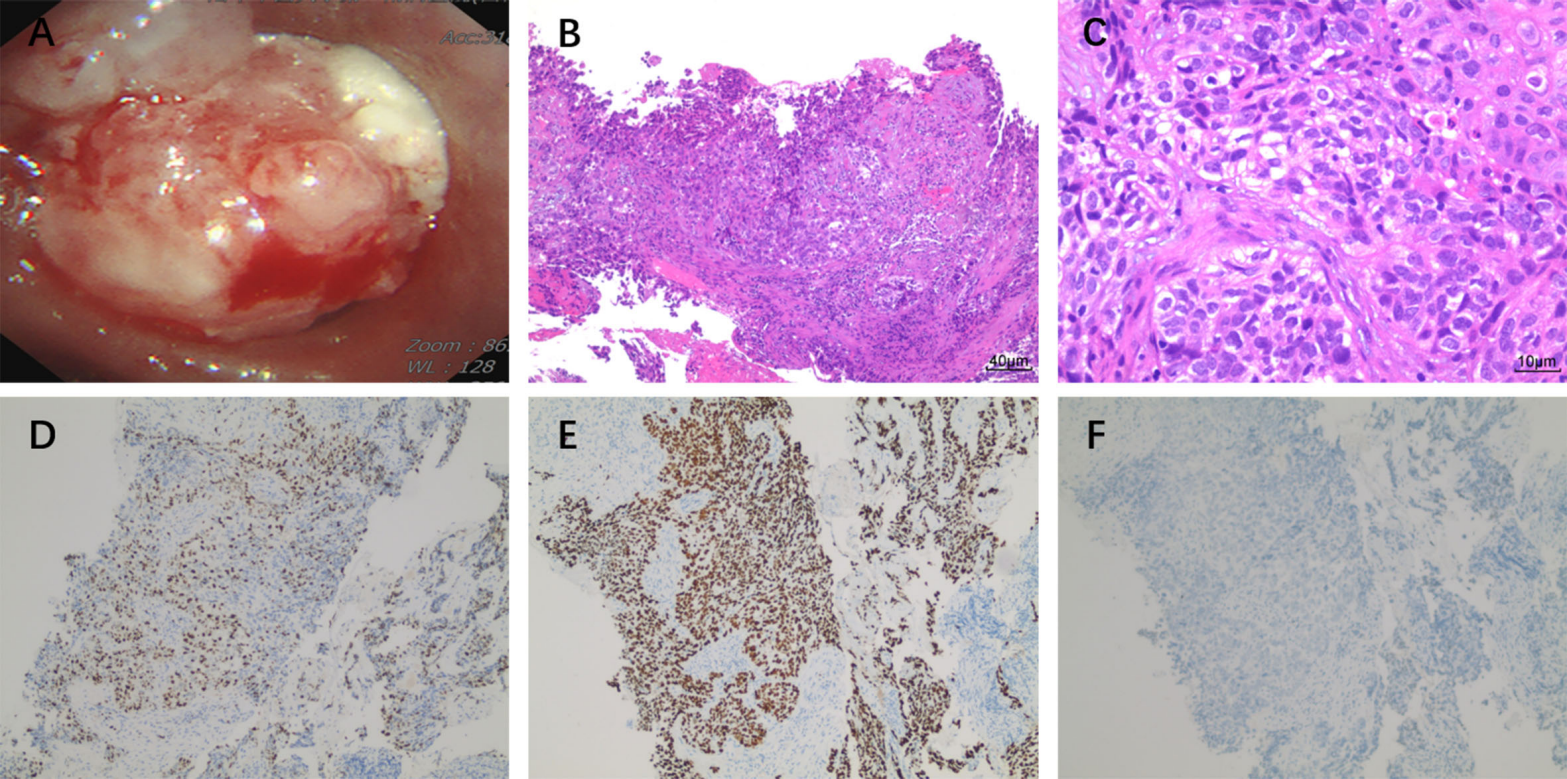
**(A)** Fiberoptic bronchoscopy shows cauliflower-like new organisms blocking the left main bronchus and oozing blood on the surface of the new organisms. The pathological classification of lung squamous cell carcinoma was confirmed by multi-biomarker immunohistochemistry. **(B)** HE×100, **(C)** HE×400, **(D)** ki-67, **(E)** P40, **(F)** TTF-1.

**Figure 2:**
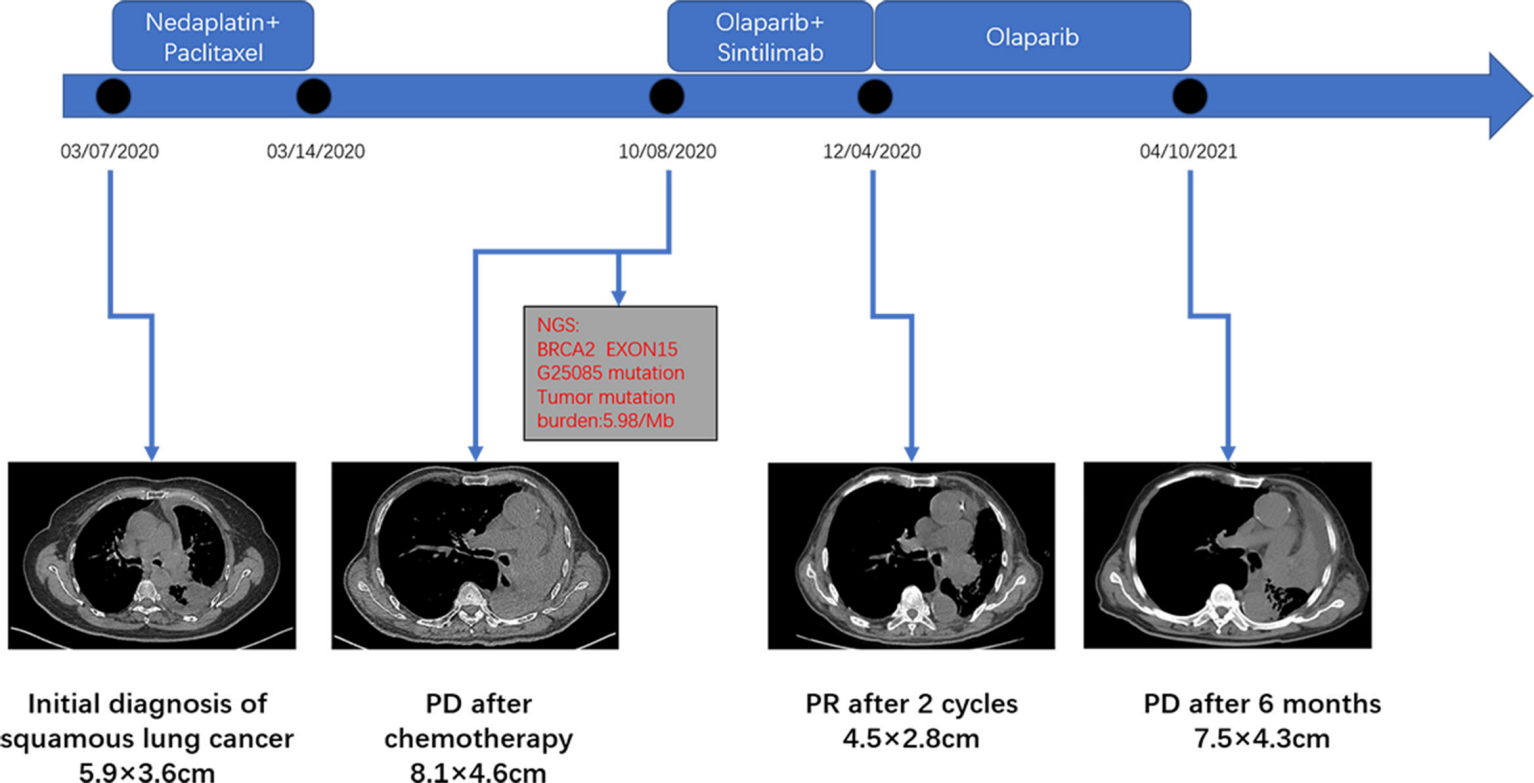
The clinical course schedule of this patient includes the treatment plan received by the patient and the chest CT of the disease response on each treatment route.

An 83-year-old male patient had no family history of hypertension, chronic obstructive pulmonary disease, or malignancy. He smoked for 40 years and had a smoking index of 20 pack-years. He was admitted to the hospital for a physical examination, during which a chest computed tomography (CT) scan revealed a left hilar occupancy measuring approximately 5.9x3.6 cm, obstructive atelectasis in the left lung field, and a small pleural effusion. Further fiberoptic bronchoscopy revealed that the left main bronchus was blocked by new organisms, the surfaces of which oozed blood. This location was the site of the biopsy, which revealed lung squamous cell carcinoma (**Figure 1A**). P40 (+), CK56 (-), ki-67 > 25%, TTF-1 (-) (**Figures 1B–F**) in immunohistochemistry. The patient ceased chemotherapy because he could not endure the adverse effects of nausea and vomiting after one cycle of perfusion chemotherapy (40 mg nedaplatin *via* bronchial artery infusion administration) and paclitaxel systemic intravenous treatment. After 7 months, the patient developed hemoptysis with a daily volume of about 10-20 mL, and chest CT scan revealed a left deviation of the mediastinum, left lung consolidation and atelectasis, a soft tissue mass in the left hilum with a size of about 8.1x4.6 cm, left main bronchial obstruction, and enlarged lymph nodes in the left cervical root and mediastinum. No obvious metastatic lesions had been found after head MRI, whole-body bone imaging, whole-abdomen enhancement CT. We took the disease’s progression into consideration (Clinical Stage, cT4N3M1a, stage IVA). Subsequently, the BRCA2 EXON15 G25085 mutation was detected by blood next-generation sequencing, and the tumor mutation load TMB was 5.98 mutations/Mb. Based on the sequencing test results, we immediately started aggressive treatment and decided to use the PARP inhibitor Olaparib (300mg twice a day) with the PD-1 inhibitor Cindilimab. After one week, the patient’s hemoptysis stopped. A repeat chest CT revealed that the left hilar mass was substantially smaller than before, with a long diameter of around 4.5 cm, and that the left lung was recruiting and the pleural effusion was absorbed after receiving Olaparib for two months and PD-1 inhibitor for two immunotherapy sessions. At this time, the patient appeared new symptoms, puffy eyelids, decreased urine volume, and generalized weakness. In further testing of common serological indicators, we found that blood creatinine increased from normal value at the beginning of the disease to 163.90μmol/L. Meanwhile, urine precipitation microscopy showed urine protein (+) without a tubular pattern. Routine blood, liver function, inflammation indicators, tumor indicators were normal, and autoantibodies were negative. Following the multidisciplinary team’s discussion, we took into account the kidney damage caused by immune checkpoint inhibitors, which was graded as 2, and stopped using the PD-1 inhibitor in accordance with the Chinese Society of Clinical Oncology (CSCO) 2022 recommendation. One month after quitting the PD-1 inhibitor, the serum creatinine level was back to normal. Six months after starting Olaparib monotherapy, the patient was more advanced. The therapy options, disease response, and PFS for each treatment line are summarized in **Figure 2**.

## Discussion

Lung cancer is still the most lethal tumor worldwide in 2022, but its incidence rate is declining year after year (10). Likely due to COVID-19 pandemic, the popularity of chest CT scan increases the detection and treatment of early lung cancer, particularly the patients with inoperable resection or distant metastases. Targeted drugs have significantly prolonged survival in lung cancer patients, especially in non-small cell lung cancer patients. Drugs specifically formulated to target the non-small cell lung cancer genes, such as EGFR, ALK, and ROS1 mutations, have already show excellent results (11, 12). With the advance of drug research and development, other rare BRAF, NTRK, RET, MET, HER2, and EGFR20 exon insertion mutations, have been also developed the corresponding targeted drugs, which have been partially approved for clinical use. Some are still in the research stage but have achieved significant efficacy (13). Several large case-control studies (2, 3, 14–16) have found that BRCA gene defects are the causative genes for seven types of cancers: hereditary breast, ovarian, prostate, pancreatic, biliary tract, esophageal, and gastric cancers. In recent years, several studies (17–19) also showed that there is a clinical correlation between BRCA2 and lung cancer. PARP inhibitors have been used successfully in clinical trials in breast and ovarian cancer patients with mutations in the BRCA gene, but no large clinical studies have been conducted to confirm whether these drugs can be used in lung cancer. So far, three case reports (20–22) showed that the PARP inhibitor Olaparib is effective in patients with BRCA1/2 mutations in non-small cell lung cancer, but all of them were lung adenocarcinoma or lung carcinoid tumors, whereas our study reported lung squamous cell carcinoma.

With the increasingly widespread use of immune checkpoint inhibitors (ICIs), there is an increasing number of adverse reactions associated with ICIs. Several large clinical studies (23, 24) confirmed that these adverse reactions can affect almost every organ and system. The most common immune-related adverse events (irAEs) include rash, vitiligo, colitis, pneumonia, pituitary inflammation, and the relatively rare ones being myocarditis, encephalitis, and renal damage. In our case report, the patient’s disease progressed after chemotherapy, and was found to have BRCA 2 positive mutation and relatively high TMB expression based on blood next-generation sequencing. BRCA1/2, and wild-type BRCA1/2 HR deficiency tumors display a higher neo-antigen load than HR-proficient cancers, producing a more effective anti-tumor immune response. In addition, there is evidence that BRCA deficiency may induce a STING-dependent innate immune response, by inducing type I interferon and pro-inflammatory cytokine production (25). Therefore, we considered the therapeutic strategy of the combinations of PARP inhibitors with immunotherapies. After communicating with the patient and family, we attempted to choose the PARP inhibitor Olaparib in combination with a PD-L1 inhibitor, and the repeat chest CT after 2 months suggested significant disease significant improvement, but the patient’s blood creatinine was elevated. Creatinine returned to normal one month after stopping PD-1, which may indicate that although targeting and immunity can help patients achieve partial response (PR) and prolong PFS, the risk of concurrent use of ICIs is significantly increased. In the study by Hu et al (26), 6220 Chinese patients with lung cancer were screened, and 1.03 percent of them had BRCA germline mutations, of which BRCA2 mutations accounted for 76.3%. In comparison to patients with typical lung cancer-driving gene mutations, patients with BRCA germline mutations tended to be younger and more likely to develop non-small cell lung cancer before the age of 50. Another retrospective analysis discovered that somatic cell mutation of the BRCA2 gene, not embryonic cell mutation, was more likely to occur in patients with non-small cell lung cancer, with a likelihood of 4.97% (27). In addition to primary breast cancer, the FDA has approved the use of PARP inhibitors in gynecologic cancers linked to germline BRCA1/2 mutations. One of the mechanisms of PARP inhibitors is based on the concept of synthetic lethality. PARP inhibitors could also be used in tumors which share molecular features of BRCA mutated tumors—known as “BRCAness”. Therefore, mutation of genes beyond BRCA in the HR pathway expands the indication of PARP inhibitors (28). The broader use of synthetic lethality targeting the HR pathway is still being investigated. Numerous preclinical trials have demonstrated that patients with the same genetic abnormalities may benefit from targeted therapy regardless of the origin of the tumor (29).The patient in our case report was up to 80 years of age at presentation and was considered to have a somatic mutation. The disease improved somewhat with the combination of PARP inhibitor Olaparib and PD-L1 inhibitor in the previous phase, but immune checkpoint inhibitor-related adverse effects occurred, and after discontinuation of PD-L1, the patient’s disease progressed after 6 months of receiving single-agent targeted therapy. This case suggests that patients with non-small cell lung cancer with BRCA2 gene mutation have a positive response to targeted therapy.

Although this study demonstrated the efficacy of Olaparib in lung squamous cell carcinoma with a BRCA2 mutation, it is not clear whether we have activated immune cells *in vivo* to inhibit tumor growth with the use of PD-L1 inhibitor in the early stage. But the blood creatinine of the patients has been in the normal range through the late follow-up and no other systemic damage occurred, the possibility of anti-tumor activity of immunotherapy is less. We will design prospective experiments in the follow-up to optimize the study conditions and collect single-agent PARP inhibitors for the targeted treatment of non-small cell lung cancer patients with BRCA mutations and provide more evidence-based evidence for the use of PARP inhibitors in non-small cell lung cancer patients with BRCA mutations.

## Data availability statement

The raw data supporting the conclusions of this article will be made available by the authors, without undue reservation.

## Ethics statement

Written informed consent was obtained from the individual(s) for the publication of any potentially identifiable images or data included in this article.

## Author contributions

ZC: writing the article and analyzing the experimental results. KW: pathological results analysis and image collection. ZC, KW, and LZ: Follow-up of medical records and immunohistochemical test. LG: plan making, publishing funds, article revision. All authors contributed to the article and approved the submitted version.
